# Supplementary material for: Iterative improvement in the automatic modular design of robot swarms
Source: PeerJ Comput Sci. 2020 Dec 7;6:e322. doi: 10.7717/peerj-cs.322 (PMC7924708; doi:10.7717/peerj-cs.322)
Supplement: Supplemental Information 3 [file peerj-cs-06-322-s003.zip › argos3/doc/api/standalone/a00475_source.html]

ARGoS: plugins/robots/foot-bot/simulator/footbot\_turret\_default\_actuator.h Source File


- Main Page
- Related Pages
- Namespaces
- Classes
- Files

- File List
- File Members

# plugins/robots/foot-bot/simulator/footbot\_turret\_default\_actuator.h

Go to the documentation of this file.

```
00001 
00007 #ifndef FOOTBOT_TURRET_DEFAULT_ACTUATOR_H
00008 #define FOOTBOT_TURRET_DEFAULT_ACTUATOR_H
00009 
00010 #include <string>
00011 #include <map>
00012 
00013 namespace argos {
00014    class CFootBotTurretDefaultActuator;
00015 }
00016 
00017 #include <argos3/plugins/robots/foot-bot/control_interface/ci_footbot_turret_actuator.h>
00018 #include <argos3/plugins/robots/foot-bot/simulator/footbot_entity.h>
00019 #include <argos3/plugins/robots/foot-bot/simulator/footbot_turret_entity.h>
00020 #include <argos3/core/simulator/actuator.h>
00021 
00022 namespace argos {
00023 
00024    class CFootBotTurretDefaultActuator : public CSimulatedActuator,
00025                                          public CCI_FootBotTurretActuator {
00026 
00027    public:
00028 
00029       CFootBotTurretDefaultActuator();
00030       virtual ~CFootBotTurretDefaultActuator() {}
00031 
00032       virtual void SetRobot(CComposableEntity& c_entity);
00033 
00034       virtual void SetRotation(const CRadians& c_angle);
00035       virtual void SetRotationSpeed(SInt32 n_speed_pulses);
00036       virtual void SetMode(ETurretModes e_mode);
00037 
00038       virtual void Update();
00039       virtual void Reset();
00040 
00041    private:
00042 
00043       CFootBotTurretEntity* m_pcTurretEntity;
00044       UInt32 m_unDesiredMode;
00045 
00046    };
00047 
00048 }
00049 
00050 #endif
```

---

Generated on 10 Jul 2018 for ARGoS by 
 1.6.1
